# Experimental Tests, FEM Constitutive Modeling and Validation of PLGA Bioresorbable Polymer for Stent Applications

**DOI:** 10.3390/ma13082003

**Published:** 2020-04-24

**Authors:** Jakub Bukala, Piotr P. Buszman, Jerzy Małachowski, Lukasz Mazurkiewicz, Kamil Sybilski

**Affiliations:** 1 Institute of Mechanics and Computational Engineering, Faculty of Mechanical Engineering, Military University of Technology, Gen. Sylwestra Kaliskiego 2, 00-908 Warsaw, Poland; jakub.bukala@wat.edu.pl (J.B.); lukasz.mazurkiewicz@wat.edu.pl (L.M.); kamil.sybilski@wat.edu.pl (K.S.); 2 Center for Cardiovascular Research and Development American Heart of Poland, Czajek 41, 40-534 Katowice, Poland; p.buszman@ahp-ccrd.org; 3 Department of Cardiology, Andrzej Frycz-Modrzewski Kraków University, Gustawa Herlinga-Grudzińskiego 1, 30-705 Cracow, Poland

**Keywords:** bioresorbable, stent, polymer, PLGA, constitutive modeling, experimental tests

## Abstract

The use of bioresorbable polymers such as poly(lactic-co-glycolic acid) (PLGA) in coronary stents can hypothetically reduce the risk of complications (e.g., restenosis, thrombosis) after percutaneous coronary intervention. However, there is a need for a constitutive modeling strategy that combines the simplicity of implementation with strain rate dependency. Here, a constitutive modeling methodology for PLGA comprising numerical simulation using a finite element method is presented. First, the methodology and results of PLGA experimental tests are presented, with a focus on tension tests of tubular-type specimens with different strain rates. Subsequently, the constitutive modeling methodology is proposed and described. Material model constants are determined based on the results of the experimental tests. Finally, the developed methodology is validated by experimental and numerical comparisons of stent free compression tests with various compression speeds. The validation results show acceptable correlation in terms of both quality and quantity. The proposed and validated constitutive modeling approach for the bioresorbable polymer provides a useful tool for the design and evaluation of bioresorbable stents.

## 1. Introduction

Heart disease is a leading cause of hospitalization and death worldwide (33% of all deaths) [1]. The dissemination of percutaneous coronary intervention (PCI) as the preferred method for revascularization (which relieves narrowing) has revolutionized the treatment of ischemic heart disease (IHD) over the past 20 years [1]. PCI is an effective and less aggravating alternative to coronary artery bypass grafting (CABG). PCI usually includes stent deployment to prevent acute recoil and vessel closure during the first months after the procedure [2]. Coronary stents are tube-shaped scaffolds designed to decompress to a certain diameter during angioplasty [3]. Metallic stents are accepted as the standard in interventional cardiology [4]. Despite high effectiveness and a relatively low complication risk, PCI is still not an ideal technique. Its main disadvantage is the implementation of a permanent metal prosthesis in the vessel wall, which can lead to potential inflammation, late-stent thrombosis, neoatherosclerosis, and restenosis [5,6].

To overcome the disadvantages of metal stents, attention has turned to the development of a bioresorbable coronary stent [7]. The term “bioresorbable” indicates that the material is naturally absorbed by the organism over time (e.g., hydrolysis and proteolytic enzymatic degradation). Bioresorption occurs when mechanical support of the artery is no longer needed, typically 12–24 months after PCI. Ultimately, the bioresorbable stent material is absorbed completely by the patient’s body and excreted as harmless metabolic waste.

Although the range of commercially available bioresorbable coronary stents is relatively small, clinical results, e.g., for Absorb™ (Abbott Laboratories) and Fantom Encore (REVA Medical) stents, show that bioresorbable coronary stents offer sufficient mechanical support for the vessel wall after PCI. Moreover, their bioresorption can reduce the risk of restenosis caused by an inflammatory reaction in the vessel wall [8].

Two groups of biomaterials are currently within the scope of research on bioresorbable coronary stents: bioresorbable polymers and biocorrosive metal alloys. Bioresorbable polymers are characterized by a fairly predictable degradation pattern, but their mechanical parameters (e.g., stiffness) are substantially lower than those of permanent stents. While biocorrosive metal alloys, such as magnesium alloys, have better mechanical properties, their degradation process is more complicated, which results in lower predictability [9]. Unpredictable stent degradation can significantly increase the risk of inflammation and, consequently, thrombosis and restenosis [10]. Thus, the stent geometry and material properties must be considered simultaneously when assessing stent performance [11], and there is a need for materials with a predictable degradation process. The currently prevailing opinion is that bioresorbable polymers are more promising in biomedical applications, especially for coronary stents. The most commonly used bioresorbable polymer is polylactide (poly(lactic acid)—PLA), a biodegradable and bioactive thermoplastic aliphatic polyester. PLA is used widely in clinical applications, e.g., bioresorbable stitches (since the 1960s), soft-tissue implants and orthopedic implants [12]. Depending on the polymerization parameters, PLA can be amorphous poly-DL-lactide (PDLLA) or heterotactic poly-L-lactide (PLLA). PLLA has relatively high crystallinity (approx. 35%) and is similar to a typical ductile polyester. PLLA is also characterized by a relatively long degradation period (approx. 2 years). By comparison, PDLLA has lower crystallinity, and hence its mechanical properties are similar to those of rubber materials, with a significantly shorter degradation period than PLLA. The mechanical properties of PLA show an anisotropic response that is highly dependent on the manufacturing process. The properties of PLA are also strain rate-dependent and highly temperature- and environment-sensitive [11].

The mechanical properties of bioresorbable polymers change under the influence of a hydrous environment (ISO 15814). These changes are strictly related to the absorption of large amounts of liquid by the amorphous fraction during the degradation process (hydrolytic degradation). For example, the mechanical properties of poly(D,L-actide-co-glycolide) (PDGLA) decline significantly after implementation (the E modulus value reaches a few percent of the initial value) [13]. In addition, the chemical composition of the medium significantly influences the rate of polymer material degradation [14]. Degradation under aqueous conditions can also be altered by the stent fabrication process, as large areas of the stent structure are affected by heat propagation during laser cutting. The high temperature gradient present during the cutting process can alter the properties of the material within the heat-affected zone, resulting in changes in the mechanical properties in terms of hydrolytic degradation compared with untreated material [15].

Finite element numerical analysis (FEA) is a valid and efficient method for investigating and optimizing the mechanical and interactive behavior of medical devices such as coronary stents [16]. Because of the complex geometry of the studied structures and the sophisticated nonlinear material properties of tissues, an advanced finite element approach is often the preferred simulation method [17]. FEA is relatively low-cost compared to experimental tests [18] and permits the determination of values that would be difficult or even impossible to obtain via empirical tests (e.g., the stress or strain rate in specific areas of the model) [19]. In addition, the ethical advantages of using FEA, which obviates the need for extensive tests on humans or animals, are significant.

Considering the potential benefits of the application of bioresorbable stents on a wider scale, it is important to develop and improve a methodology for FEA of bioresorbable stents [20]. The key problem is to model the behavior of bioresorbable polymers [21]. Any progress in this field would significantly increase the probability of developing new bioresorbable coronary stents that could improve the treatment and quality of life of the millions of patients suffering from coronary artery disease worldwide. Considering the vast range of dependences, it is crucial to properly describe the mechanical behavior of PLA in relation to the exact application [12].

In [10], an advanced Bergstrom-Eswaran material model for the simulation of bioresorbable polymers is presented. The model consists of two parallel branches: one representing hyperelasticity (Langevin theory) and the other representing visco-plastic effects. It is a highly complicated model that requires a significant number of material constants. A similar approach is presented in [12], in which the anisotropic and visco-plastic behavior of PLLA is modelled by two parallel molecule networks. The first network consists of an isotropic Neo-Hooke model with visco-plastic yield after exceeding the activation energy (stress based), while the other network implements anisotropic hyperplastic relations, which represent the material behavior under large strain in different directions. A different approach is presented in [22] and [23], where simplified constitutive modeling of bioresorbable polymers is shown. The simplified modeling methodology is similar to that typically used for metallic materials and represents isotropic elastic-plastic behavior. In this approach, material stiffening (isotropic/kinematic) is also implemented as a function of plastic strain. The advantage of this approach is its relatively simple implementation with satisfactory representation for bioresorbable polymers. The main disadvantage is the lack of a strain rate or anisotropic representation [24].

Few papers have described a constitutive modeling methodology for bioresorbable polymers such as PLA. Moreover, there is a huge gap between the complex approaches in [8,10], which require a wide range of material constants, and the relatively simple approach presented in [22]. This gap suggests the need for a compromise approach that combines simplicity of implementation and strain rate dependence.

## 2. Materials and Methods

### 2.1. Experimental Tests of the PLGA Bioresorbable Polymer

**Figure 1:**
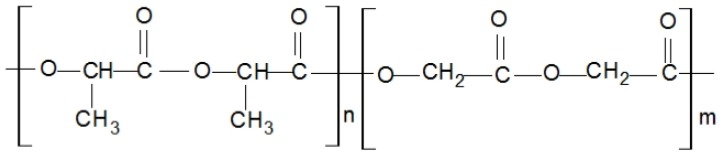
Chemical formula of the poly(lactic-co-glycolic acid) (PLGA) bioresorbable polymer developed at the Centre of Polymer and Carbon Materials [27].

Two series of experimental tests of poly(lactic-co-glycolic acid) (PLGA) and PLLA blend bioresorbable polymers were performed to quantify the mechanical response of the material. The considered material was originally developed at the Centre of Polymer and Carbon Materials (CPCM) of the Institute of the Polish Academy of Sciences [25,26]. It is a copolymer of lactic acid and glycolic acid (the chemical formula is presented in Figure 1, while the basic physical-chemical properties, based on data provided by CPCM, are presented in Table 1) [27].

Tensile tests of this material were performed using Kappa 50 DS Materials Testing Machines (Ulm, Germany) by Zwick and Roel, which were additionally equipped with an INSTRON thermal chamber. The material properties were investigated at a temperature of 38.0 °C, which represents typical conditions for in vivo coronary stent decompression.

Before the tests, all specimens were stored in a sealed package (to prevent the penetration of water vapor) in a refrigerator at a temperature of approx. 7 °C. Each specimen set was investigated on the same day that the package was opened, and the specimens were removed from the package immediately before placement in the thermal chamber. The thermal chamber also covered the tensile machine grips. The thermal chamber was turned on 2 h before the tests to ensure a uniform temperature in the chamber and the tensile machine grips.

The physical dimensions of the specimens were measured individually prior to the placement in the thermal chamber. After mounting in one grip of the machine, each specimen was held without movement for 15 min before the test started and was then mounted in the second grip.

The specimens were in the form of tubes extruded with a stent manufacturing extruder. The parameters of the specimens were, in general terms, identical to those of tubes obtained in the manufacturing process of commercial bioresorbable stents made of PLGA/PLLA (stent production using laser cutting technology).

The aim of the test study is to obtain a tensile force vs. machine grip displacement curve representing the global stiffness as well as the local deformation process. Ten specimens in the form of tubes (outer diameter d_pz_ = 1.8 mm, wall thickness t = 150.0 µm, specimen length l_c_ = 90.0 mm) made of PLGA/PLLA were considered. The dimensions of the specimens were measured using an optical microscope equipped with dedicated image processing software (DIC).

**Figure 2:**
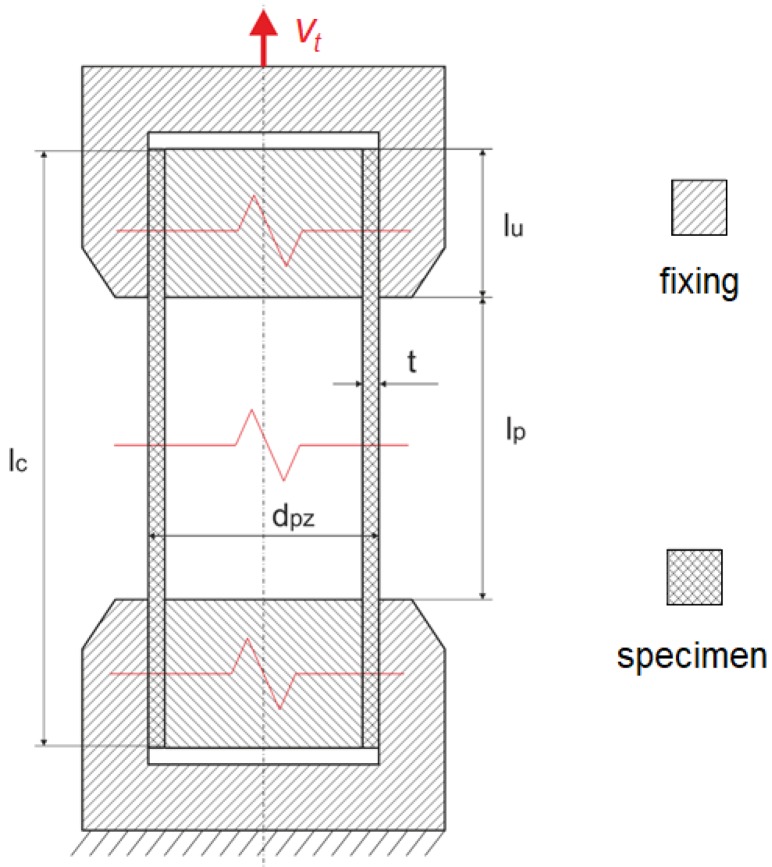
Schematic overview (cross section) of mounting of tubular specimens in the tensile test of the PLGA bioresorbable polymer (longitudinal tension).

Metal cylinders (l_u_ = 25.0 mm, diameter dependent on the tube inner diameter) were inserted in the ends of the specimens to prevent crushing of the specimens and to enable proper fixing in the machine grips (Figure 2). Therefore, the gauge length of each specimen was l_p_ = 40.0 mm. The elongation process was assumed to be equal to 100%, and hence the displacement was equal to 40.0 mm.

To verify the influence of the strain rate, tests were performed at 2 strain rates: 0.0003 1/s and 0.03 1/s. These values were based on numerical simulations of the typical stent implementation procedure [28] (Table 2).

### 2.2. Constitutive Modeling

General elastic-plastic material was adopted for the purpose of this study. Material anisotropy was not modeled in this case. The focus was on a strain rate modeling strategy as well as on kinematic/isotropic hardening. Therefore, an isotropic elastic-visco-plastic model was used. In this approach, the hardening function $f_{h}\left(\varepsilon_{eff}^{p}\right)$ is described as a tabular (piecewise) or linear function [29], where $\varepsilon_{eff}^{p}$ is the effective plastic strain:

(1) $$\varepsilon_{eff}^{p}=\overset{t}{\underset{0}{\int }}{\left(\frac{2}{3}{\dot{\varepsilon }}_{ij}^{p}{\dot{\varepsilon }}_{ij}^{p}\right)}^{\frac{1}{2}}dt$$

and the plastic strain rate ${\dot{\varepsilon }}_{ij}^{p}$ is the difference between the total strain rate and the elastic strain rate:

(2) $${\dot{\varepsilon }}_{ij}^{p}={\dot{\varepsilon }}_{ij}-{\dot{\varepsilon }}_{ij}^{e}$$

The yield function is defined by:

(3) $$\phi =\frac{1}{2}s_{ij}s_{ij}-\frac{\sigma_{y}^{2}}{3}\leq 0$$

where $\sigma_{y}$ is the yield stress (scalar value):

(4) $$\sigma_{y}\left(\varepsilon_{eff}^{p},{\dot{\varepsilon }}_{eff}^{p}\right)=\beta \left({\dot{\varepsilon }}_{eff}^{p}\right)\left[\sigma_{0}+f_{h}\left(\varepsilon_{eff}^{p}\right)\right]$$

$\beta \left({\dot{\varepsilon }}_{eff}^{p}\right)$ is a strain rate-dependent factor, and $\sigma_{0}$ is the initial yield strength.

Model implementation in a discrete approach is realized by iteratively updating the stress tensor ($s_{ij}$) and checking the yield stress ($\sigma_{y}$). When the yield stress criterion is met, the stress tensor is accepted; otherwise, the plastic strain value $\varepsilon_{eff}^{p}$ is increased, and the stresses are relaxed back to the updated yield surface. An iterative algorithm is used to find the correct plastic strain increment $\Delta \varepsilon_{eff}^{p}$ by solving the root of the following equation [30,31]:

(5) $$f\left(\Delta \varepsilon_{eff}^{p}\right)={\left(\frac{3}{2}s_{ij}^{*}s_{ij}^{*}\right)}^{\frac{1}{2}}-3G\Delta \varepsilon_{eff}^{p}-\sigma_{y}\left(\varepsilon_{eff}^{p}+\Delta \varepsilon_{eff}^{p},\frac{\Delta \varepsilon_{eff}^{p}}{\Delta t}\right)$$

The secant iteration is applied to find the root (*k*—iteration subscript):

(6) $$\Delta \varepsilon_{eff}^{p,k+1}=\Delta \varepsilon_{eff}^{p,k}-f\left(\Delta \varepsilon_{eff}^{p,k}\right)\frac{\Delta \varepsilon_{eff}^{p,k}-\Delta \varepsilon_{eff}^{p,k-1}}{f\left(\Delta \varepsilon_{eff}^{p,k}\right)-f\left(\Delta \varepsilon_{eff}^{p,k-1}\right)}$$

with the starting values $\Delta \varepsilon_{eff}^{p,0}=0$ and $\Delta \varepsilon_{eff}^{p,1}=\Delta \varepsilon_{eff}$ (purely plastic increment).

When the plastic strain increment $\Delta \varepsilon_{eff}^{p}\;$ is established, the deviatoric stress is scaled back:

(7) $$s_{ij}^{n+1}=\frac{\sigma_{y}\left(\varepsilon_{eff}^{p}+\Delta \varepsilon_{eff}^{p},\frac{\Delta \varepsilon_{eff}^{p}}{\Delta t}\right)}{{\left(\frac{3}{2}s_{ij}^{*}s_{ij}^{*}\right)}^{\frac{1}{2}}}s_{ij}^{*}$$

Visco-plastic behavior is realized in the following way (Cowper-Symonds model) [29]:

(8) $$\beta =1+{\left(\frac{\dot{{\dot{\varepsilon }}_{eff}^{p}}}{C}\right)}^{\frac{1}{P}}$$

The tangent stiffness matrix is consistently derived from this integration scheme.

Material constants, i.e., the strain rate influence parameters *C* and *P*, are presented in Table 3, while the tabular description of the stress-effective yield strain relation is presented in Table 4. The listed constant values were determined using correlation methods (least squares method) in the MATLAB environment based on the experimental results.

### 2.3. Model Verification and Validation

Model verification was carried out based on the longitudinal tensile tests, while model validation was performed based on independent bioresorbable stent compression tests. In the first case, numerical analyses represented the experimental tests described in Section 2.1. The experimental results were then compared with the numerical results of analogous simulations. In the second case, the tests focused on determining stent radial forces (compression forces) as a function of compression diameters for newly cut (undeformed) stents. The experimental results were then compared with the numerical results of analogous simulations. The use of undeformed stents in the tests allowed for the comparison of the exact (within the range of manufacturing precision) geometries in both the experimental and numerical tests.

The simulated problems (especially compression of a bioresorbable coronary stent) were characterized with all types of nonlinearities recognized in FEA, namely, large deformations (geometric nonlinearities), nonlinear material properties (physical nonlinearities) and contact modeling (boundary condition nonlinearities) [29]. Consequently, nonlinear computations are necessary, and the adopted computational scheme was nonlinear implicit (iterative and incremental) [32].

A free stent compression procedure in a 3D domain involves slight stent displacements and rotational movements. Therefore, the defined boundary conditions do not meet the requirements of typical static analyses. In order to enable a proper simulation of this issue, additional dynamic parts were included in the FEA basic equation [29,33]:

(9) $$M{\ddot{d}}_{n+1}+D{\dot{d}}_{n+1}+\left[K_{t}\left(d_{n}\right)+K_{c}\left(d_{n}\right)\right]\Delta d_{0}=P{\left(d_{n}\right)}_{n+1}-F\left(d_{n}\right)-F_{c}\left(d_{n}\right)$$

where *M* is the mass matrix, *D* is the damping matrix, $d_{n}$ is the nodal displacement vector, ${\dot{d}}_{n+1}$ is the nodal velocity vector in time n+1, ${\ddot{d}}_{n+1}$ is the nodal acceleration vector in time n+1, $K_{t}$ is the nonsingular stiffness matrix, $K_{c}$ is the contact stiffness matrix, $\Delta d_{0}$ is the displacement increment, $P{\left(d_{n}\right)}_{n+1}$ is the nodal forces vector in time n+1 (based on deformation in time *n*), $F\left(d_{n}\right)$ is the unbalanced forces vector in time *n*, and $F_{c}\left(d_{n}\right)$ is the contact forces vector.

Although the analysis is still considered quasi-static, the ratio of kinematic and damping energy to total energy is insignificant (less than 1%) because of the relatively long simulation times [33].

Equation (5) was solved using quasi-Newton (Broyden-Fletcher-Goldfarb-Shanno updates) as well as Newmark-*β* (dynamic parts) schemes [34]. Relative linear displacements and strain energy for a given increment were the convergence norms. The numerical solver used in this case was LS-DYNA^®^ developed by Livermore Software Technology Corp. [29] (Livermore, CA, USA). The software provides both explicit and implicit computation schemes. The double-precision version solver as well as computation using massively parallel processing were adopted (implicit computation scales well in terms of computation time depending on the number of adapted CPUs) [29].

#### 2.3.1. Longitudinal Tensile Tests

In the numerical analyses, the model geometry reflected the average of the measured dimensions of all test specimens (Table 5).

**Figure 3:**
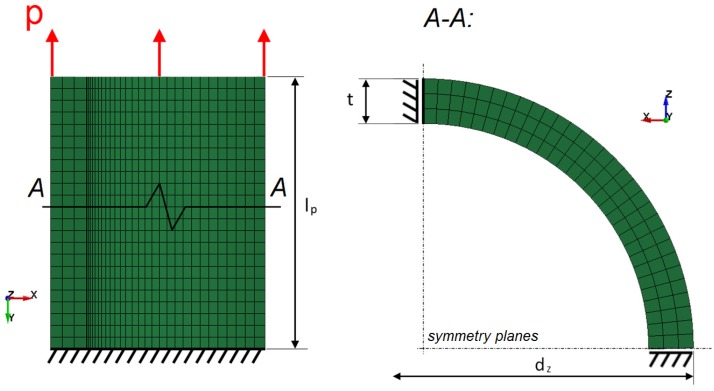
FEM model of a specimen tube in the longitudinal tensile test: symmetry boundary conditions are marked in black, and the displacement load is marked in red.

Because of the assumed symmetry of the specimen and its boundary conditions, the test was modelled at 1/4 of the geometry with the use of additional boundaries (Figure 3).

Model discretization was based on 8-node elements formulated as fully integrated solids. The quantity of elements was equal to 72,000 (finite element characteristic dimensions; the mesh density was determined in mesh sensitivity studies for more complex models; see Section 2.3.2).

Boundary conditions were defined as fully constrained nodes at one end of the tube, two symmetry planes (YZ and XY), and the kinematic (displacement) load at the other end of the tube. The load velocity values for particular variants were identical to those presented in Table 2.

Numerical modeling and simulation of the experimental tests were performed by implementing the constitutive modeling strategy presented in Section 2.2.

#### 2.3.2. Stent Compression Tests

Three sets of specimens in the form of newly cut out (undeformed) stents were examined. The specimens, which were identical to those used in the tests presented in Section 2.1, were cut out from the tubes made of PLGA bioresorbable polymer. The specimens were cut out by the stent manufacturer (Balton^®^, Apollo, Warsaw, Poland) using a picosecond laser device and the stent geometries provided. The geometries were identical to those used in the numerical simulations (considering the manufacturing correction for cutting). The dimensions of the tube and final stents (specimens) are presented in Table 6.

**Figure 4:**
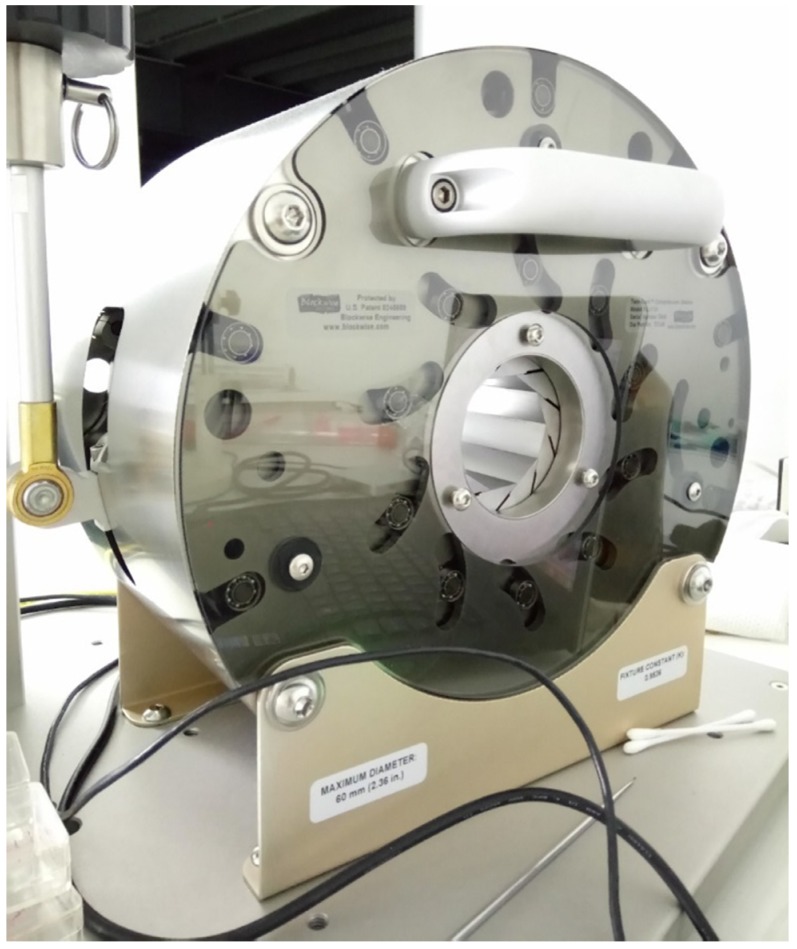
Crimping machine used in the stent compression tests. The machine was equipped with a radial force measuring head (friction compensated) and a thermal chamber.

The tests were performed using a specially equipped laboratory crimping machine dedicated to stent compression (BLOCKWISE RLU, Large Twin-Cam™, Figure 4; owned by Balton^®^, Balton Ltd., Warsaw, Poland). Special equipment included a measuring head, which registered the compensated radial force as a function of time or compression diameter, and a thermal chamber that permitted the maintenance of a constant temperature of 38 °C (the temperature of the tests presented in Section 2.1) inside the test area.

Test series were performed in three variants corresponding to different compression times and consequently, different compression rates, as shown in Table 7.

**Figure 5:**
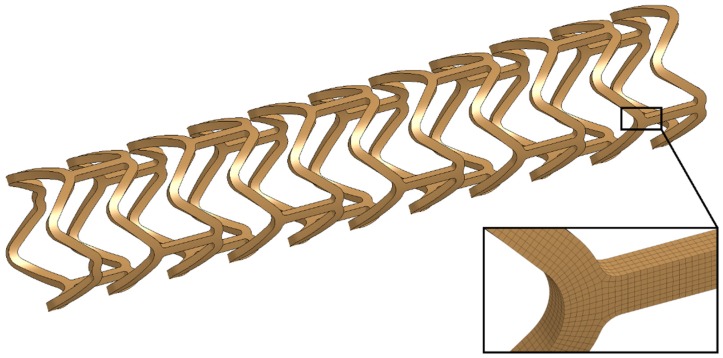
Model geometry for validation analyses of the PLGA bioresorbable polymer constitutive model.

Numerical modeling and simulations of the experimental tests were performed by implementing the constitutive modeling strategy presented in Section 2.2. The model geometry (Figure 5) and the subsequent discrete model were developed based on the cutting path used for manufacturing the specimens presented in Section 2.3.1.

Stent geometry was discretized with the use of 259536 elements formulated as fully integrated 8-node solids [34]. A mesh sensitivity study was performed considering four variants of mesh density, namely, 3, 5, 7, and 9 elements along the bending surface of the stent struts. Based on the obtained results and previous experience, a 7-element variant was selected as a suitable compromise between discretization accuracy and demand for computational resources (time, memory) [35].

**Figure 6:**
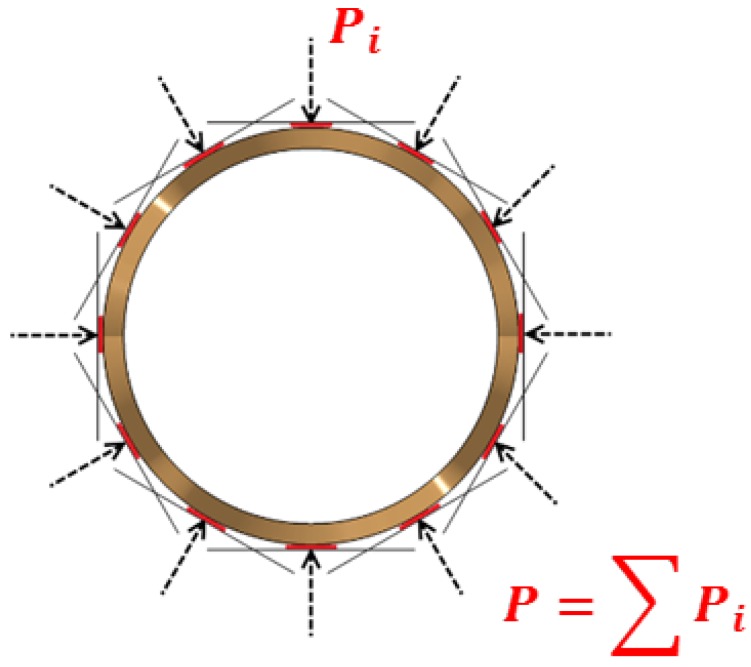
Boundary conditions for free stent compression: front view of the displacement of 12 predefined rigid wall structures (shown in black).

The boundary conditions for free stent compression included the displacement of 12 predefined rigid wall structures representing the kinematics of the crimping machine head (Figure 6). Rigid wall displacement was described as a function of position over time according to the parameters presented in Table 7.

The contact mechanism was defined by a penalty formulation method in which the FE code introduces normal interface springs between all nodes penetrating the contact surface. To improve the convergence of the calculation, the Mortar segment-to-segment contact option as well as an IGAP parameter of 10.0 (used to stiffen the contact for large penetrations without affecting moderate penetrations) were implemented [29]. The friction model in the Mortar contact is a standard Coulomb friction law expressed in terms of the tangential contact stress, with a static friction coefficient *µ* = 0.05 [36].

### 2.4. Biological Reponse in the Large Model of Coronary In-Stent Restenosis

The part of study was approved by local bioethical committee for animal experimentation (Medical University of Silesia, Katowice, Poland). To validate the mathematical and mechanical modeling tests described above, three PLGA/PLLA blend polymer stents (3.0 in diameter, 15.0 mm length) were implanted with standard percutaneous techniques in three coronary arteries of domestic swine with 110% overstretch. Following coronary angiography, intravascular optical coherence tomography (OCT) was utilized before and after the stent implantation to size the vessel. Stents were deployed after reaching body temperature with slow in mid portion of one of the three coronary arteries with stepwise inflation—2 atmospheres per 5 s. Following post implantation coronary angiography and OCT were performed. Animals were scheduled for 28-day follow-up with coronary angiography and OCT.

## 3. Results

### 3.1. Numerical vs Experimental Modeling

**Figure 7:**
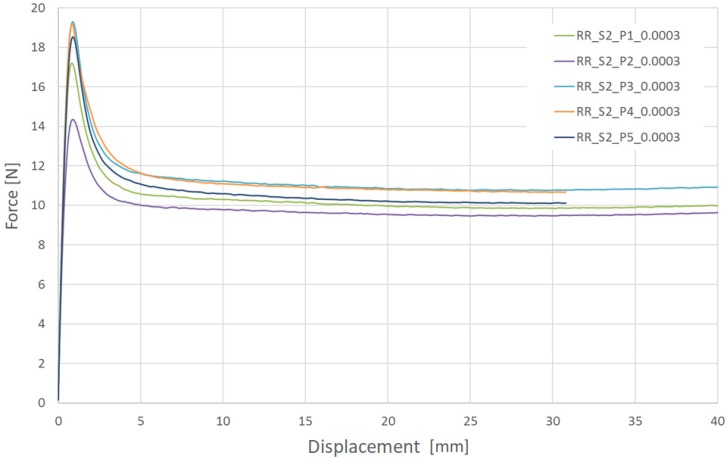
Longitudinal tensile force vs machine grip displacement for tubular specimens made of PLGA (assumed strain rate 0.0003 1/s).

**Figure 8:**
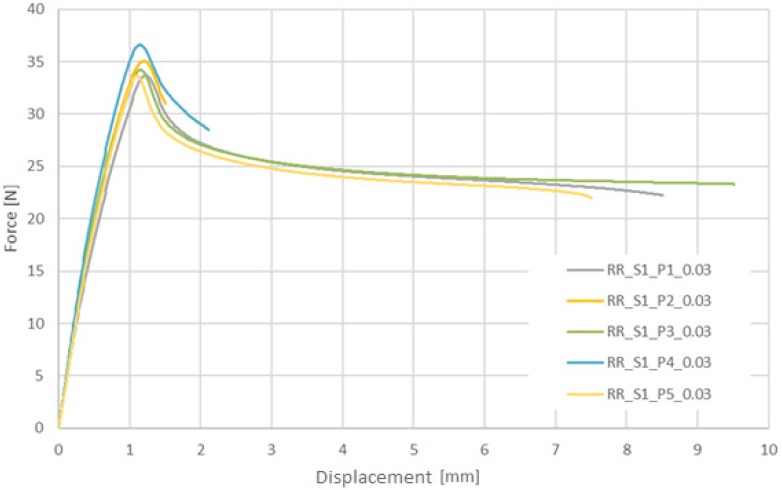
Longitudinal tensile force vs machine grip displacement for tubular specimens made of PLGA (assumed strain rate 0.03 1/s).

In Figure 7 and Figure 8, the results of two series of experimental tests of PLGA are presented for particular displacement speed variants. The speed was 0.72 mm/min at a strain rate of 0.0003 1/s and 72.0 mm/min at 0.03 1/s.

**Figure 9:**
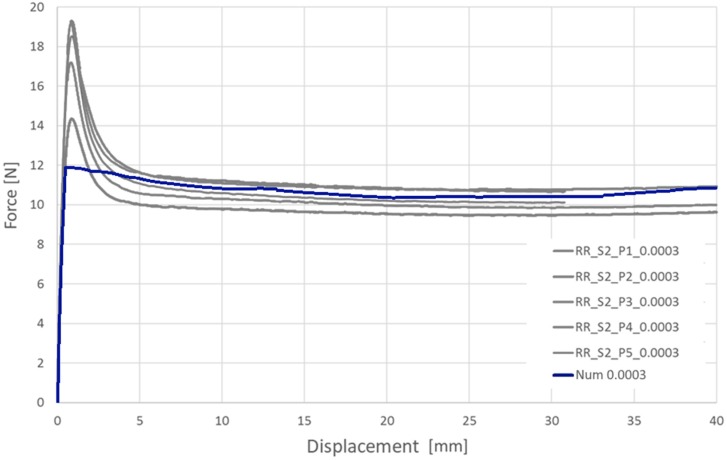
Comparison of the experimental and corresponding numerical results of tension forces during longitudinal tensile tests of tubes made of PLGA bioresorbable polymer: variant 1:0.72 mm/min.

**Figure 10:**
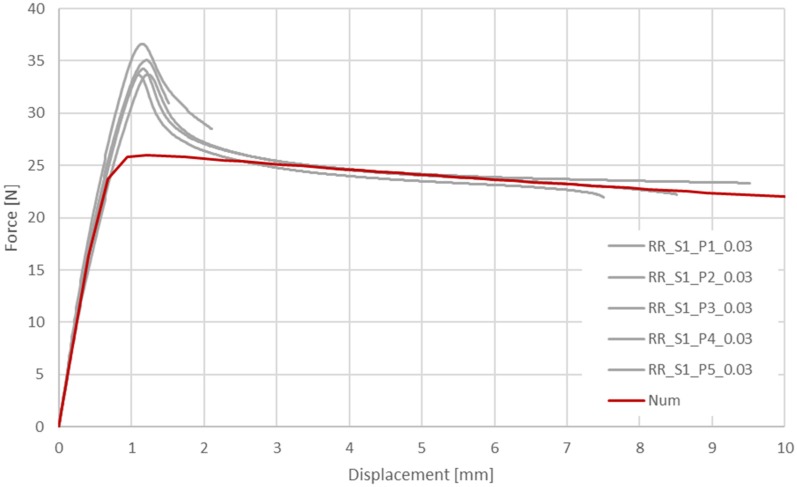
Comparison of the experimental and corresponding numerical results of tension forces during longitudinal tensile tests of tubes made of PLGA bioresorbable polymer: variant 2:72.0 mm/min.

The experimental and numerical results are compared in Figure 9 and Figure 10.

**Figure 11:**
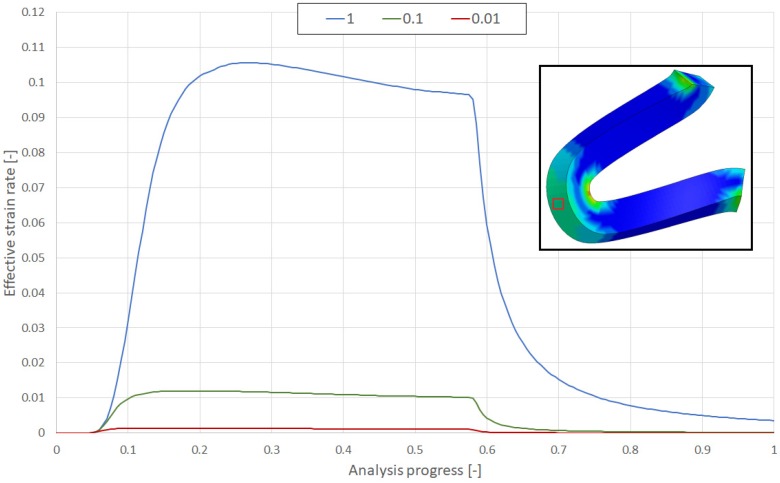
Effective strain rates for 3 compression rate variants: 1.0 mm/s (blue), 0.1 mm/s (green), and 0.01 mm/s (red).

The results of numerical analyses of the effective strain rates for different variants for the so-called “knee-area,” which is the area that undergoes the greatest deformation, are shown in Figure 11.

**Figure 12:**
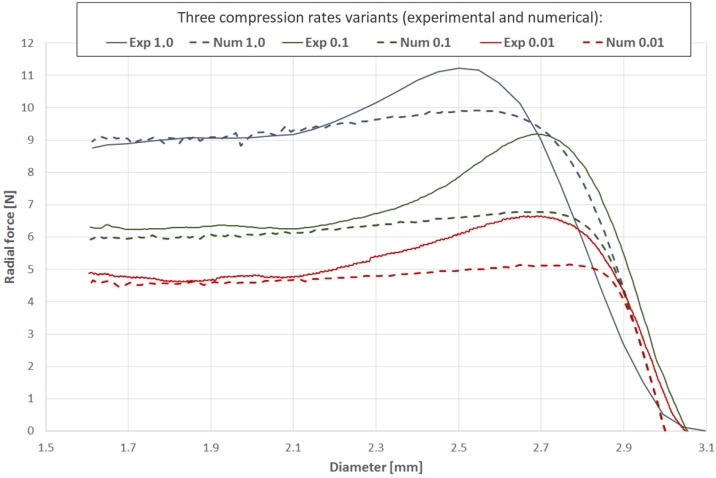
Comparison of experimental and numerical results for radial forces during free compression of undeformed coronary stents made of PLGA bioresorbable polymer with three compression rate variants: 1.0 mm/s, 0.1 mm/s, and 0.01 mm/s.

The experimentally recorded radial force and the corresponding numerical results are compared in Figure 12. The compression radial force was calculated throughout the course of the analysis as the sum of rigid wall normal forces (contact forces between rigid walls and the stent outer surface, Figure 10) [35].

### 3.2. Biological Response

**Figure 13:**
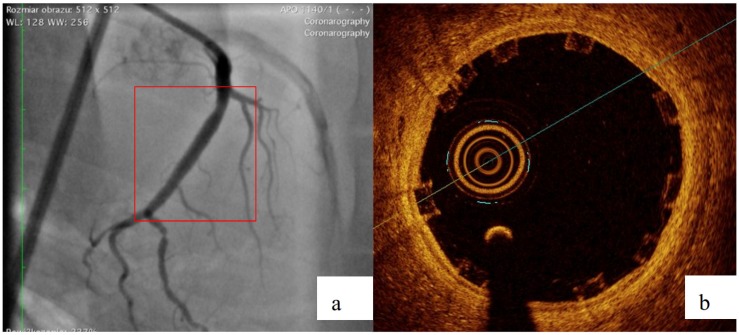

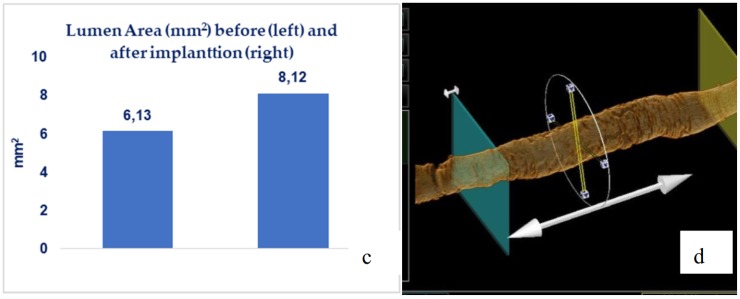
Acute outcome after PLGA/PLLA blend polymer stent implantation in coronary angiography (**a**) and optical coherence tomography (**b**). Lumen area increase before and after implantation (**c**). Geometrical view of vessel after stent implantation (**d**).

**Figure 14:**
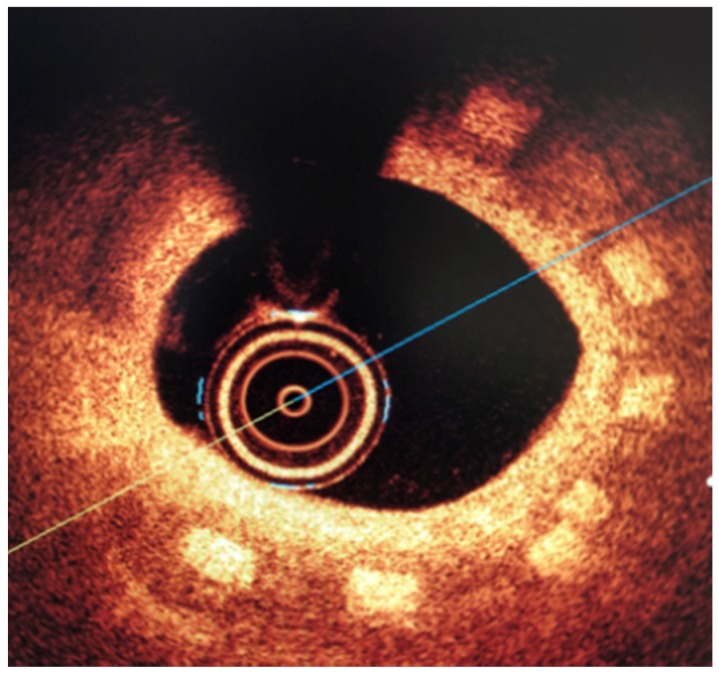
Optical coherence tomography at 28 days follow-up show optimal vascular response and stent geometry. Stent struts show blurred image, a sign of early hydrolysis (rectangle).

All three stents were implanted successfully. In coronary angiography stents showed enough radial force to support overstretched vessel (Figure 13). The OCT has shown good stent apposition and the analysis has shown lumen enlargement post implantation 24% as shown in Figure 13c. At 28-day follow-up, all stents were covered with neointima, and struts embedded. Interestingly the signs of material hydrolysis were observed thus proving the polymer amorphic properties (Figure 14).

## 4. Discussion

The investigated material behavior was similar to that of ductile materials, especially other thermoplastic polymers. Elastic behavior with a relatively stable (constant) elastic stiffness (elastic modulus) was clearly visible at small elongation values. With further elongation, a decrease in force was observed around the yield, as found in other thermoplastic polymers. However, the scale of the phenomenon was especially significant in the investigated case (the force decrease range was 35–50% in relation to the peak value). After force stabilization, relatively stable plastic-deformation behavior was observed until break. During this phase, the measured force remained approximately unchanged, suggesting limited hardening of the material (considering the necking of specimens).

Among the ten tested specimens, only three remained intact until the end of the test (40.0 mm of displacement/approx. 100% elongation). Considering a strain rate of 0.03 1/s, most of the specimens were broken in a range of less than 3 mm, corresponding to an elongation of approximately 5%. In the case of a strain rate of 0.0003 1/s, three specimens remained intact until the end of the test, and the other two were broken at approximately 30.0 mm of displacement, corresponding to approximately 75% elongation.

Taking into consideration the presented results (force-displacement charts for both variants), it can be concluded that the examined material is sensitive to the strain rate. Higher strain rates increased the stress curve courses as well as yield stress values but significantly decreased the elongation obtained at break. In practice, these results indicate that bioresorbable stent compression and the implementation procedure need to be performed over a relatively long period of time to ensure small strain rates in the stent structure.

After the yield stress, the observed strength decreased significantly (30–50%). The magnitude of this decrease was also related to the specific strain rate. Similar behavior is observed for some thermoplastics, which suggests a visco-related phenomenon related to polymer network properties (chain breaking/new chain reorganization). Following the decrease in yield strength, a rather stable course was observed. In this range, the material behaved in a way that can be described as plastic with kinematic hardening.

The value spread observed in the present tests is definitely unfavorable, especially the values of elongation observed at break. Thus, the examined material is highly sensitive to manufacturing conditions (potential discontinuities and, hence, stress concentrations as well as local and subsequent global damage).

Verification of the results via longitudinal tensile tests and validation of radial forces showed an acceptable correlation, in terms of quality and quantity, between the experimental and numerical plots (relative difference of final values for particular variants of less than 5%). Particularly important is the relatively good correlation of the results at high values of strain, which are crucial for stent performance evaluation.

The differences in the initial diameters and elastic range properties (elastic modulus) are due to inevitable deviations of the manufacturing process, i.e., limitations of extruding and cutting precision.

The results presented in the mathematical modeling has shown sufficient radial force, inflate, expand and implant a tested stents in the coronary vessel. This was validated by mechanical radial force tests as well as implantation in the in-vivo, porcine in-stent restenosis model. The implanted stents provided sufficient strength to withheld the compression of on oversized vessel. The struts were well apposed. At long-term follow-up the stent areas remained similar, thus confirming time-dependent tensile and radial strength. This pivotal study showed optimal neointimal coverage of all stents struts which were embedded without excess of neointima. Additionally, the struts in OCT were absorbable to light from OCT, in contrary to immediate effect after implantation. This could be the result of hydrolysis, and amorphous character of the tested polymer. This requires however further investigation of the explanted material.

## 5. Conclusions

The bioresorbable PLGA polymer material considered here is characterized by relatively low stiffness (especially compared with metal alloys or even more crystal polymers), strain rate sensitivity, a significant decrease in observed strength after yield, and plastic behavior with limited hardening. Therefore, the investigated material is complex and problematic in terms of constitutive modeling and simulation.

The literature review revealed the need for a constitutive modeling strategy for bioresorbable polymers that combines simplicity of implementation with the most important material characteristics, namely, strain rate dependency and a plasticity model with hardening.

The crucial point of constitutive modeling in terms of the intrinsic material behavior representation is the local strain characterization. The authors have tried to address the local strain measurement problem, however, it was not possible because of technical limitations of the tests performed in the thermal chamber (problems with efficient camera observation). In the future research, this issue is going to be undertaken in order to investigate the local response, particularly the cross-sectional area tracking. The authors have also performed the tensile tests of the discussed material using standardized specimens. In this case, the material for specimens was casted, contrary to the tube material formed (extruded) as a final product. The results were characterized with the following aspects: breaking strains were unrepeatable with significant deviation; the peak stress values compared to the plastic range were characterized with a high descent (almost two times). Therefore, the authors believe that the use of tubular specimens, identical to those obtained in the manufacturing process of commercial bioresorbable stents made of PLGA, is crucial even though it prevents the use of the local strain measurement. In that case, it was also proved that the global response of the material is more important than the local measurements if the global response is sought. Additionally, the global response of the material was obtained using the most suitable specimens and then verified and validated in two separate experimental/numerical comparisons.

The proposed constitutive modeling approach for the bioresorbable polymer is based on an isotropic elastic-visco-plastic model (Cowper-Symonds). Validation of the methodology based on two separate experimental tests confirmed strain rate dependency (Figure 11) and a plasticity model with hardening, key mechanisms of PLGA behavior. For all considered variants, the obtained force results (Figure 12) showed an acceptable correlation between the numerical and experimental curves, proving the accuracy of the adopted approach.

Using FEA and the proposed constitutive model, it is possible to perform numerous case analyses with reasonable modeling accuracy. Therefore, this model is a useful tool for both the design and evaluation of bioresorbable stents. Numerical methods cannot replace the use of experimental tests, but they are capable of illustrating certain trends and dependences at almost every stage of stent development.

**Figure 15:**
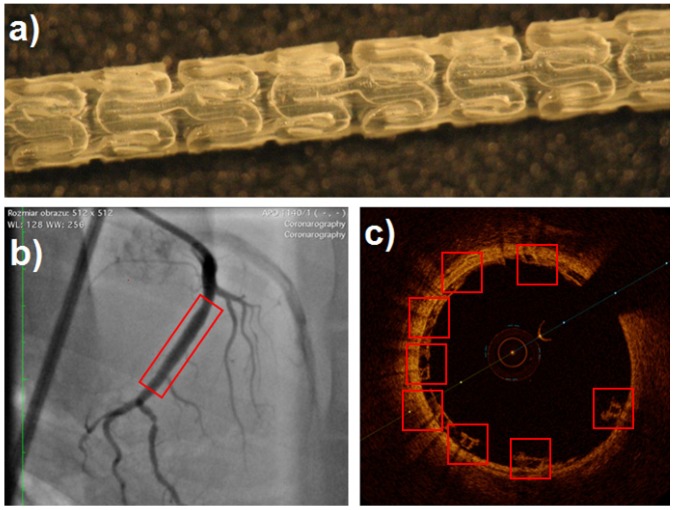
One of the test variants of a bioresorbable coronary stent developed with the use of the proposed constitutive methodology within the Apollo STRATEGMED2 project: (**a**) Stent after cutting and free compression; (**b**) area of clinical test implementation (angiography image); (**c**) area of clinical test implementation (intravascular ultrasound image).

The presented constitutive methodology was successfully used in the design process of bioresorbable stents within the Apollo STRATEGMED2 project. An exemplary stent prototype is presented in Figure 15.

## Figures and Tables

**Table 1:** Basic physical-chemical properties of the PLGA polymer provided by CPCM [25].

Characteristic/Property of the PLGA Material	Value
Appearance:	light yellow to pale brown
Form:	pellet (3.0–6.0 mm in diameter)
Polymer composition:	lactic acid: 82–88% (based on the results of 1H NMR ^1^ analysis)
Weight average molar mass:	180000.0–300000.0 g/mol (according to GPC ^2^ (polystyrene))
Inherent viscosity:	1.8–3.1 dL/g (0.1% in chloroform, 25.0 °C)
Residual monomers:	below 1.4% mass (measured in 1H NMR)
Residual content of organic solvents:	below 50 ppm
Glass transition temperature:	52.0–60.0 °C
Softening point temperature:	153.0–159.0 °C
Heat of melting:	above 40.0 J/g
Young’s modulus at 25.0 °C:	2400.0 ± 160.0 MPa
Tensile strength at 25.0 °C:	70.0 ± 1.0 MPa
Young’s modulus at 38.0 °C:	2000.0 ± 160.0 MPa
Tensile strength at 38.0 °C:	50.0 ± 1.0 MPa

^1^ Proton nuclear magnetic resonance/NMR spectroscopy with respect to hydrogen-1 nuclei; ^2^ Gel permeation chromatography/size-exclusion chromatography (SEC).

**Table 2:** Kinematic assumptions for a longitudinal tensile test of PLGA tubular-type specimens.

Variant	Assumed Strain Rate ^1^ $\dot{\varepsilon }$ (1/s)	Grip Displacement Speed (Figure 2) V_t_ (mm/min)
1	0.0003	0.72
2	0.03	72.0

^1^ strain rate for a given variant calculated in relation to the initial sample length.

**Table 3:** Material constants of the applied constitutive model of the PLGA bioresorbable polymer.

Young’s Modulus *E* (MPa)	Poisson Ratio *ν*	Yield Stress *σ_y_* (MPa)	Density *ρ* (kg/m^3^)	Strain Rate Parameter *C*	Strain Rate Parameter *P*
1800.0	0.45	12.0	1100.0	1 × 10^−5^	3.0

**Table 4:** Material constants of the applied constitutive model of the PLGA bioresorbable polymer.

	1	2	3	4	5	6	7	8
Plastic strain $\varepsilon_{eff}^{p}$	0.0	0.2	0.4	0.6	0.9	1.2	1.5	2.0
Effective stress $\sigma_{eff}^{}$ (MPa)	12.0	13.8	17.0	21.8	34.1	64.7	122.3	314.3

**Table 5:** Model specimen dimensions in numerical longitudinal tensile tests (Figures 2 and 5).

Tube Wall Thickness *t* (mm)	Tube Outer Diameter *d_pz_* (mm)	Tube Gauge Length *l_p_* (mm)
0.15	1.80	40.0

**Table 6:** Specimen nominal dimensions.

Tube/Stent Outer Diameter *d_pz_* (mm)	Tube Wall Thickness *t* (mm)	Stent Length *l_c_* (mm)
3.00	0.15	15.0

**Table 7:** Kinematic assumptions for radial compression tests of stent specimens made of PLGA.

Variant	Compression Time *t_c_* (s)	Compression Rate *c_r_* (mm/s)	Initial Diameter *d_i_* (mm)	Compressed Diameter *d_c_* (mm)	No. of Specimens
1	1.4	1.0	3.0	1.65	2
2	14.0	0.1	3.0	1.65	2
3	140.0	0.01	3.0	1.65	2
